# Editorial: Inflammatory factors in coronary heart disease: mechanism, diagnosis, and therapy

**DOI:** 10.3389/fcvm.2023.1234132

**Published:** 2023-09-25

**Authors:** Qian Dong, Zhiyang Li, Qingwei Ji, Kunwu Yu

**Affiliations:** ^1^ Department of Cardiology, Union Hospital, Tongji Medical College, Huazhong University of Science and Technology, Wuhan, China; ^2^ Department of Cardiology, The People’s Hospital of Guangxi Zhuang Autonomous Region, Nanning, China

**Keywords:** coronary heart disease, inflammatory factors, mechanism, diagnosis, therapy

**Editorial on the Research Topic** Inflammatory factors in coronary heart disease: mechanism, diagnosis and therapy

Coronary heart disease (CHD), one of the main causes of death in humans, is a group of clinical syndromes caused by the progression of coronary atherosclerosis. The effectiveness of conventional drugs or invasive treatments has reached a bottleneck. In recent years, studies have defined coronary heart disease as an inflammatory disease. Inflammatory factors, including anti-inflammatory factors and pro-inflammatory factors, have gradually become important biomarkers and effective targets for the treatment of coronary heart disease.​ In this Research Topic, titled “Inflammatory Factors in Coronary Heart Disease: Mechanism, Diagnosis, and Therapy”, we received 24 submissions and accepted 11 manuscripts. In addition, two abstracts were also included. Among the articles, two of the research papers explored the role and molecular mechanisms of drug or inflammation-related genes in animal models, four studies are about the correlation analysis between inflammation-related factors and ischemic cardiomyopathy or CAD, one study is about the bifurcation strategies using second-generation drug-eluting stents, and three articles are meta-analysis or reviews.

Wei et al. found that edgeworthia gardneri (Wall.) Meisn. (EG) extract protects against myocardial infarction by inhibiting NF-κB-and MAPK-mediated endothelial inflammation, indicating EG as a potential therapeutic agent in ischemic cardiovascular disease (Wei et al.). Furthermore, Zuo et al. established a remote ischemic postconditioning (RIPostC) rat model and revealed by transcriptome analysis that RIPostC could markedly reduce infarct size and decrease the level of myocardial pro-inflammatory factors, including IL-1β and IL-6, but that it can increase the level of IL-10, which was negatively correlated with ADAMTS15 (Zuo et al.). The authors point out that the role and the mechanism of the inflammation-related gene ADAMTS15 should be investigated. In another study, Wang et al. identified five potential biomarkers (SERPINA3, FCN3, PTN, CD163, and SCUBE2) in human ischemic cardiomyopathy (ICM) via datasets, and performed the functional experiment on ICM rats. These studies are expected to provide clinicians with useful tools for ICM diagnosis and treatment from the perspective of inflammation (Wang et al.).

Another study calculated the mean platelet volume lymphocyte ratio (MPVLR) in patients with chronic total occlusion (CTO) and analyzed the relationship between MPVLR and coronary collateral circulation (CCC) formation (Niu et al.). The results revealed that MPVLR was negatively correlated with CCC, and a high MPVLR level was an independent predictor of poorly formed CCC, which constitutes a simple and practical method for diagnosis. Xiong et al. revealed that sLAG3 levels in patients with coronary heart disease were significantly lower than those in the control group, and sLAG3 levels were negatively correlated with the occurrence of coronary heart disease but not with the severity of coronary heart disease. At the same time, sLAG3 was negatively associated with BMI and diabetes, suggesting that sLAG3 reduction may be a new risk factor for CAD (Xiong et al.). Another clinical study included all cardiovascular disease (CVD) patients who participated in the National Health and Nutrition Examination Survey (NHANES 2011–2014); serum albumin was measured, revealing a J-shaped association between low serum albumin levels and increased long-term mortality of CVD, which may provide a simple method for assessing the risks of CVD in the general population of the United States (Li et al.).

Cha et al. conducted a cohort study of clinical outcomes in Korean patients with diabetes using a bifurcation strategy with a second-generation drug-eluting stent; a total of 905 patients with DM and marked bifurcation lesions were enrolled in the study. The primary outcome was the 5-year incidence of target lesion failure (TLF), which was defined as a composite indicator of cardiac death, target vessel myocardial infarction, and target lesion revascularization. The results demonstrated that the T- or V-stenting technique but not the crush or culotte technique in patients with DM resulted in increased TLF compared to the one-stent strategy (Cha et al.).

In addition, research hotspots, frontiers, and development trends in anti-inflammatory studies for coronary heart disease over the past 30 years were summarized, showing that a total of 5,818 articles focused on anti-inflammatory studies in CHD (Zhang et al.), which is of great significance for future studies. Another meta-analysis, by Liu et al. includes a total of 23 studies about CRP and MACE and suggests that CRP is a prospective predictor of prognosis in patients with AMI undergoing PCI, especially in those who are hospitalized, have a short-term prognosis, and those from Asian descent (Liu et al.). In China, Danhong injection (DHI) is recommended by expert consensus and is widely used in the perioperative management of patients with acute coronary syndrome (ACS). A systematic review and meta-analysis by Li et al. demonstrated that Danhong injection combined with conventional treatment has a better therapeutic effect on patients with ACS than conventional treatment alone by inhibiting inflammation (Li et al.). Kawasaki disease (KD) is an acute, inflammation-mediated vasculitis that primarily affects children under 5 years old and is considered the most common coronary artery disease in children. A series of studies have identified vascular endothelial cell damage and dysfunction in patients with KD. Qiu et al. systematically described the role of endothelial cells in the pathogenesis of KD and the therapeutic methods of endothelial cells (Qiu et al.).

In summary, the 11 articles included in this Research Topic cover multiple themes, such as the cellular and molecular mechanisms of inflammatory factors in CHD, the influence of inflammatory factors on CHD, and inflammatory factors as biomarkers of CHD, as well as a review about the clinical outcomes targeting inflammatory factors in the treatment of coronary heart disease.

